# Engaging Isatins and Amino Acids in Multicomponent One-Pot 1,3-Dipolar Cycloaddition Reactions—Easy Access to Structural Diversity

**DOI:** 10.3390/molecules28186488

**Published:** 2023-09-07

**Authors:** Hua Zhao, Yufen Zhao

**Affiliations:** Institute of Drug Discovery Technology, Qian Xuesen Collaborative Research Center of Astrochemistry and Space Life Sciences, Ningbo University, Ningbo 315211, China

**Keywords:** multicomponent reactions, 1,3-dipolar cycloadditions, spiro-oxindole, isatin, one-pot reactions

## Abstract

Multicomponent reactions (MCRs) have undoubtedly emerged as the most indispensable tool for organic chemists worldwide, finding extensive utility in the synthesis of intricate natural products, heterocyclic molecules with significant bioactivity, and pharmaceutical agents. The multicomponent one-pot 1,3-dipolar cycloaddition reactions, which were initially conceptualized by Rolf Huisgen in 1960, find extensive application in contemporary heterocyclic chemistry. In terms of green synthesis, the multicomponent 1,3-dipolar cycloaddition is highly favored owing to its numerous advantages, including high step- and atom-economies, remarkable product diversity, as well as excellent efficiency and diastereoselectivity. Among the numerous pieces of research, the most fascinating reaction involves the utilization of azomethine ylides generated from isatins and amino acids that can be captured by various dipolarophiles. This approach offers a highly efficient and convenient method for constructing spiro-pyrrolidine oxindole scaffolds, which are crucial building blocks in biologically active molecules. Consequently, this review delves deeper into the dipolarophiles utilized in the 1,3-dipolar cycloaddition of isatins and amino acids over the past six years.

## 1. Introduction

Multicomponent reactions (MCRs) are a captivating field of chemical synthesis that have captured the attention of researchers worldwide. MCRs involve the simultaneous engagement of three or more components, resulting in products that incorporate the elements of all starting materials in their frameworks in one pot [1,2,3,4]. This distinctive characteristic renders MCRs an exceptionally powerful tool for generating novel molecular structures with unparalleled diversity. One of the most appealing characteristics of MCRs is their capacity to rapidly enhance molecular diversity. By combining multiple reactants in a single step, chemists can create complex molecules with ease and efficiency. This offers synthetically robust strategies for step-economic diversification, enabling scientists to explore a wide range of novel molecular frameworks [5,6,7,8,9,10]. Despite substantial progress in MCR research, the concurrent formation of multiple chemical bonds and stereocenters in a single step continues to pose a daunting task for chemists. MCRs represent an exciting frontier in contemporary chemistry, which have immense potential for advancing our understanding of chemical synthesis and unlocking novel avenues for drug discovery and other applications.

In recent years, there has been a growing interest in the synthesis of heterocyclic compounds using methods that are both step-economical and environmentally friendly [11,12,13,14,15,16,17,18]. One such method that has emerged as particularly useful is the 1,3-dipolar cycloaddition (1,3-DC) reaction. First pioneered by Huisgen in the 1960s [19,20,21], this reaction involves the addition of a triatomic 4π electron chemical substance to a dipole, typically an alkene or alkyne, resulting in the construction of five-membered heterocycles. The versatility and efficiency of 1,3-dipolar cycloaddition have established it as one of the most favored methods for synthesizing heterocyclic compounds [22,23,24,25,26,27,28,29,30,31,32,33,34,35]. This groundbreaking reaction has unlocked new possibilities for synthesizing tetrasubstituted pyrrolidine derivatives with multiple stereocenters from azomethine ylides and activated alkenes in a single step. However, despite its immense potential, this reaction still presents challenges that require attention. One of the challenges faced is to enhance control over selectivity in terms of regioselectivity and diastereoselectivity. 

**Figure 1:**
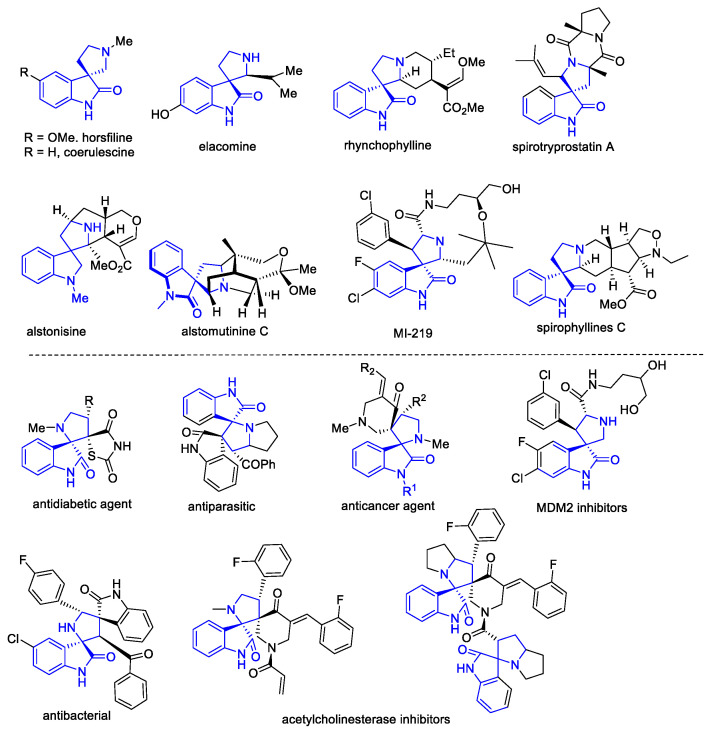
Representative natural products and bioactive compounds with the spiropyrrolidine oxindole scaffolds.

Spiro-oxindole motifs are widely recognized as pivotal components in natural products and biologically active molecules. These unique structural features provide a remarkable level of conformational rigidity, often resulting in enhanced biological activity for various biomolecules. In fact, the spiro-oxindole fusion has been demonstrated to impart unique biological properties upon several indole alkaloids and related synthetic compounds. As illustrated in Figure 1, the spiro-oxindole scaffold serves as the core structure for natural products with complex structures such as horsfiline [36], elacomine [37], rhynchophylline [38], spirotryprostatin A [39], alstomutinine C [40], and MI-219 [41]. Furthermore, the prevalence of spiro-oxindole motifs in nature highlights their significance as fundamental building blocks for drug discovery. Spiropyrrolidine oxindole moieties are not only naturally occurring, but they can also be found in a wide range of structures that exhibit impressive pharmacological activities, including antidiabetic, antiparasitic, anticancer, MDM2 inhibitor, and antibacterial properties (Figure 1) [42,43,44,45]. Given their significant importance in the field of medicine and healthcare, it is unsurprising that these valuable structures have garnered considerable attention from researchers around the world [46,47,48,49,50]. An innovative approach involves multicomponent 1,3-dipolar cycloadditions of azomethine ylides through the decarboxylative condensation of α-amino acids with isatins, which has emerged as a practical tool for creating new spiropyrrolidine oxindole moieties with even more potent therapeutic effects [51,52,53]. Noticeably, natural amino acids are abundant and environmentally benign feedstocks for synthetic chemistry. At the same time, isatins possess a unique structure consisting of an indole ring fused with a ketone group, which endows them with remarkable chemical and biological properties [54,55,56,57,58,59,60,61]. Hence, the utilization of both amino acids and isatins effectively facilitates the synthesis of a diverse and complex library of spiropyrrolidine oxindole compounds.

**Figure 2:**
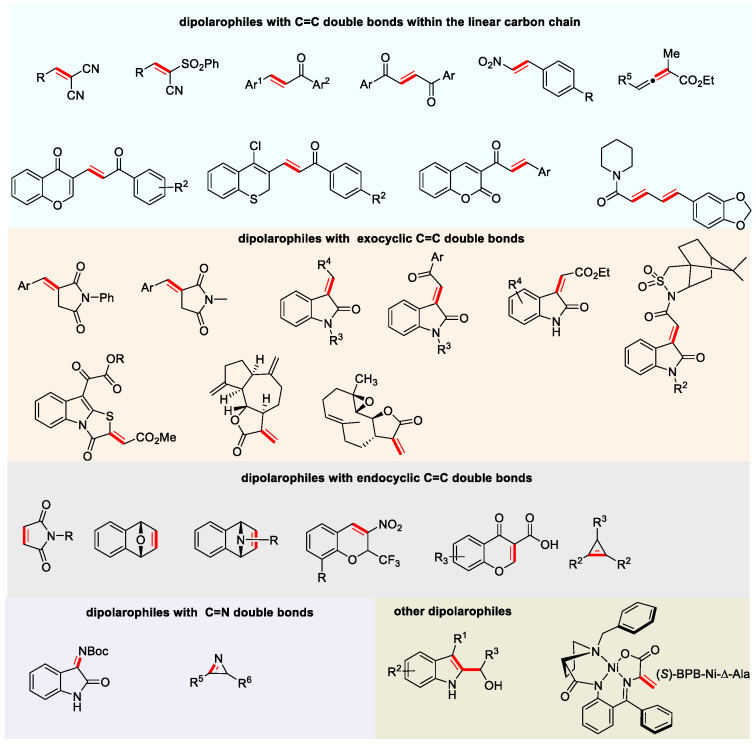
The various dipolarophiles toward 1,3-dipolar cycloaddition reactions of azomethine ylides derived from isatins and amino acids.

This concise yet informative minireview explores the fascinating realm of intermolecular one-pot three-component 1,3-dipolar cycloaddition reactions involving azomethine ylides derived from isatins and amino acids, in conjunction with a diverse array of unsaturated groups serving as dienophiles. This approach offers a multitude of advantages, including environmentally friendly synthesis, accessibility, cost-effectiveness, diversity, and sustainability. The review is succinctly organized based on the structural types of dipolarophiles, encompassing those with C=C double bonds within the linear chains, exocyclic C=C double bonds, endocyclic C=C double bonds, and C=N double bonds. (Figure 2). The review is mainly focused on sources in the literature published from 2017 to 2023.

## 2. The Dipolarophiles Containing C=C Double Bond Location within the Linear Carbon Chains

**Scheme 1:**
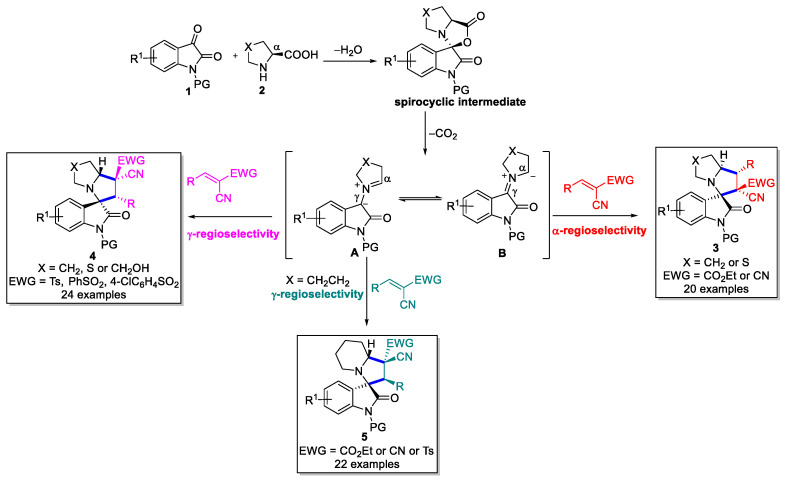
Substrate-controlled regioselectivity switch in a three-component 1,3-dipolar cycloaddition reaction.

In 2022, a novel and structurally complex series of 3,3′-pyrrolidinyl-spirooxindoles derivatives **3**–**4** containing four contiguous and two quaternary stereogenic centers were synthesized via the additive-free 1,3-dipolar cycloaddition reaction of isatin-derived azomethine ylides with α-cyano-α, β-unsaturated compounds [62]. The desired products were obtained in high yields (up to 92%) and excellent diastereoselectivities (up to >25:1 dr). The regioselectivity of this reaction was found to be influenced by the steric effects of substrates, resulting in either α- or γ-regioselectivity (Scheme 1). The remarkable versatility of pipecolic acid as a substrate for this reaction is evident, as it consistently yields compound **5** regardless of variations in electron-withdrawing groups, such as ester, cyano, or tosyl (Ts) (22 examples).

**Scheme 2:**
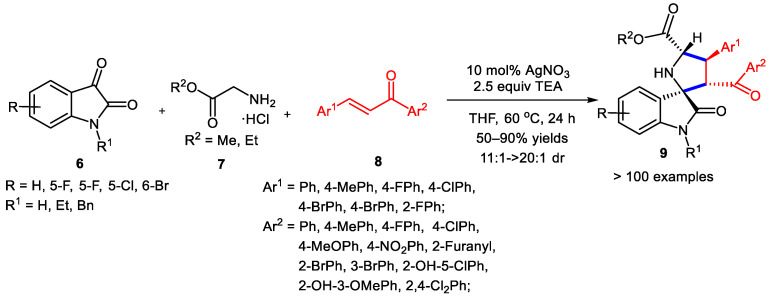
Ag-catalyzed [3 + 2] cycloaddition of substituted isatins and primary α-amino acid esters with chalcones.

Previous studies have reported the use of amino acids, benzylamine [63,64], and trifluoroethylamine [65,66,67] as reagents for generating azomethine ylides with isatins. However, there are limited reports on the utilization of stabilized azomethine ylides such as α-amino acid esters in three-component 1,3-dipolar cycloaddition reactions. In 2018, Lu et al. developed a Ag-catalyzed [3 + 2] cycloaddition reaction of azomethine ylides generated in situ from the condensation of substituted isatins **6** and primary α-amino acid esters **7** with chalcones **8** (Scheme 2). This facile and efficient method enabled the synthesis of spiroquaternary stereocenters fused in a single ring structure, resulting in products **9** containing four consecutive stereocenters. The reaction proceeded smoothly under mild conditions, affording moderate to high yields (50–95%) of the desired products. Moreover, the versatility of these products was demonstrated through a series of functional group transformations including reduction, oxidation, hydrolysis, and amidization. It is worth mentioning that this methodology can be successfully applied to a wide range of substrates (>100 examples) [68]. 

**Scheme 3:**
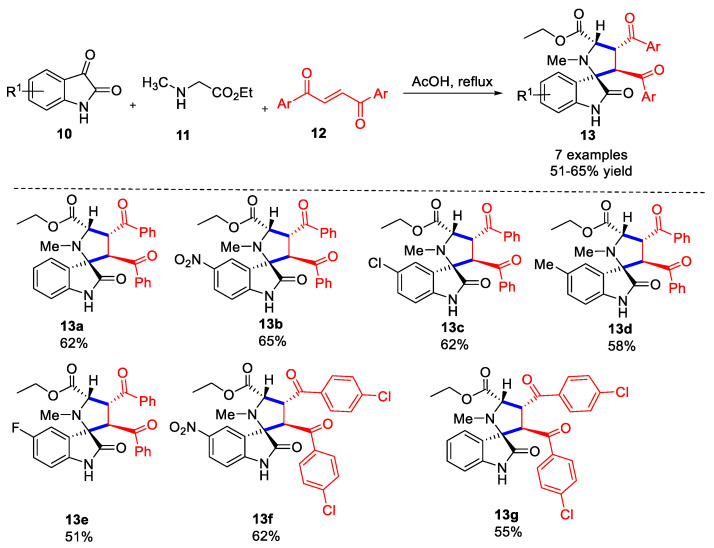
Three-component one-pot reaction using *trans*-1,2-dibenzoylethylene 12 as the dipolarophile.

In 2021, Quiroga et al. achieved a one-pot sequential three-component synthesis of novel spiro[indoline-pyrrolidine] derivatives **13** using *trans*-1,2-dibenzoylethylene **12** as the dipolarophile (Scheme 3). The reactions proceeded via a 1,3-dipolar cycloaddition between azomethine ylides generated in situ from the condensation of isatins **10** with sarcosine ethyl ester **11** under reflux conditions. This protocol offered mild reaction conditions and operational simplicity, while providing high regioselectivity and stereoselectivity. The regio- and stereochemical outcomes of this cycloaddition reaction were determined through spectroscopic analysis. The geometries and electronic structures of the resulting compounds were further investigated using DFT quantum chemical calculations, aiming to establish a comprehensive understanding of the experimental results [69].

**Scheme 4:**
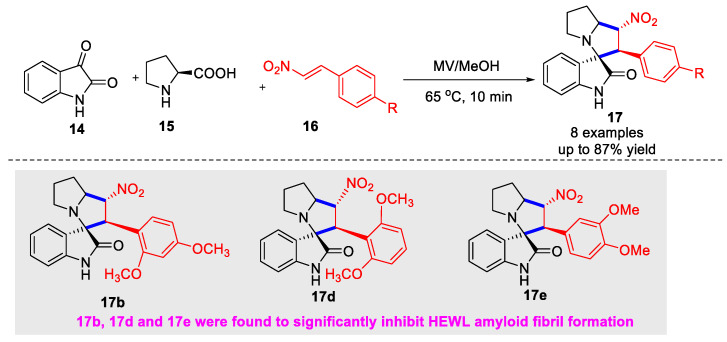
Microwave-assisted rapid synthesis of spiro-oxindole–pyrrolizidine analogues and their activity as anti-amyloidogenic agents.

In 2021, the Maniam group successfully demonstrated a microwave-assisted, one-pot, three-component 1,3-dipolar cycloaddition of azomethine ylides derived from L-proline and isatin with various β-nitrostyrenes **16** (Scheme 4). The resulting nitro-sox compounds (**17b**, **17d** and **17e**) were found to significantly inhibit HEWL amyloid fibril formation by up to 55.4%, as determined by ThT studies. After conducting further investigations, it was discovered that the potential activity of these promising candidates against amyloid misfolding, a phenomenon associated with Alzheimer’s disease pathology, was supported by various methods such as MTT assay, Raman spectroscopy, TEM, and molecular docking [70].

**Scheme 5:**
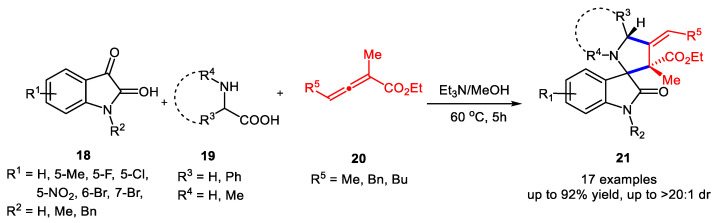
The three-component reaction involving α,γ-dialkylallenoate esters, isatin derivatives, and amino acids.

In 2017, a three-component reaction was employed to synthesize novel polycyclic spiropyrrolidine oxindoles **21** featuring two vicinal quaternary centers and a unique exocyclic C=C double bond (Scheme 5). The utilization of α,γ-dialkylallenoate esters **20**, isatin derivatives **18**, and amino acids **19** in an endo-selective 1,3-dipolar cycloaddition process resulted in impressive high yields and diastereoselectivities reaching up to 92% yield and >20:1 dr [71].

**Scheme 6:**
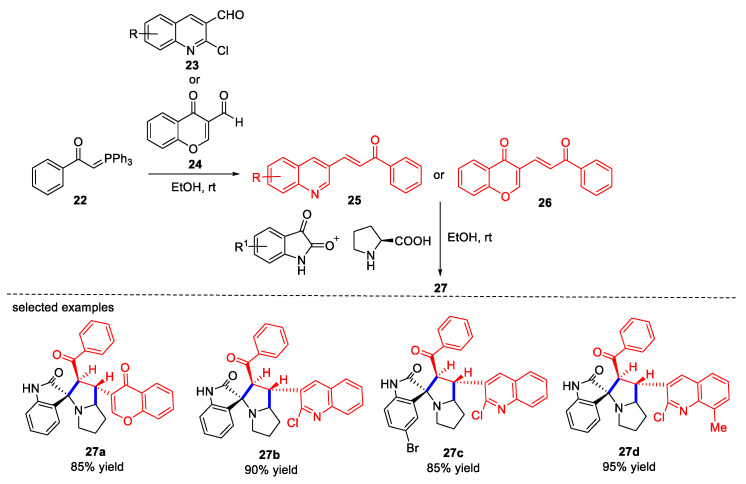
Four-component regio- and diastereoselective synthesis of pyrrolizidines incorporating spiro-oxindole/indanedione via 1,3-dipolar cycloaddition reaction.

In 2019, Halvagar et al. reported an efficient and highly selective method for synthesizing a diverse library of polycyclic pyrrolizidine-fused spiro-1,3-indandione/oxindole derivatives in EtOH at room temperature (Scheme 6). This was achieved through a one-pot sequential four-component reaction involving the in situ generation of azomethine ylides by condensing isatin and L-proline with 1-phenyl-2-(1,1,1-triphenyl-*λ*^5^-phosphanylidene)ethan-1-one) **22**, 2-chloroquinoline-3-carbaldehyde **23**/4-oxo-4*H*-chromene-3-carbaldehyde **24**. The protocol demonstrated excellent chemical yields, and high diastereoselectivity [72].

**Scheme 7:**
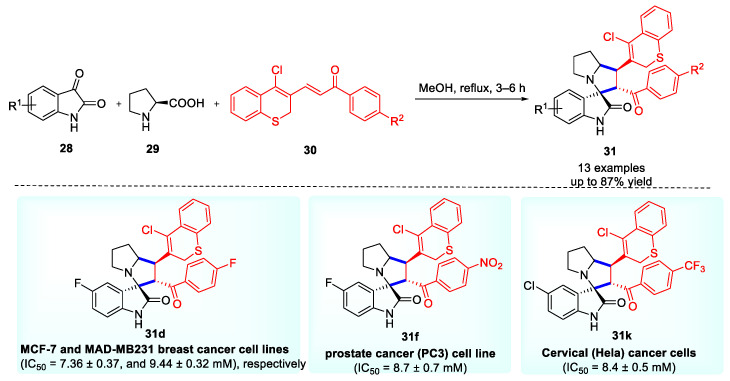
Synthesis of a series of spiro-oxindole pyrrolidine-grafted thiochromene scaffolds as potential anticancer agents.

In 2021, Barakat reported the condensation of substituted isatin derivatives with L-proline to generate azomethine ylides. These ylides subsequently underwent three-component 1,3-dipolar cycloaddition reactions with chalcones based on a thiochromene scaffold. This reaction produced a series of new spiro-heterocycles engrafted with spiro-oxindole/pyrrolidine/thiochromene scaffolds **31** in a fully controlled regio- and stereo-selective manner (Scheme 7). Importantly, promising results as good candidates for further studies were obtained from anti-cancer assays. For the breast cancer MCF-7 cell lines, compound **31d** exhibited an IC_50_ value of 7.36 ± 0.37 µM, while it also demonstrated greater activity against the MDA-MB231 breast cancer cell line with an IC_50_ value of 9.44 ± 0.32 µM. Compounds **31f** demonstrated greater potency against PC3 (IC_50_ = 8.7 ± 0.7 µM), whereas hybrid **31k** was most active against cervical cancer HeLa (IC_50_ = 8.4 ± 0.5 µM) [73]. 

**Scheme 8:**
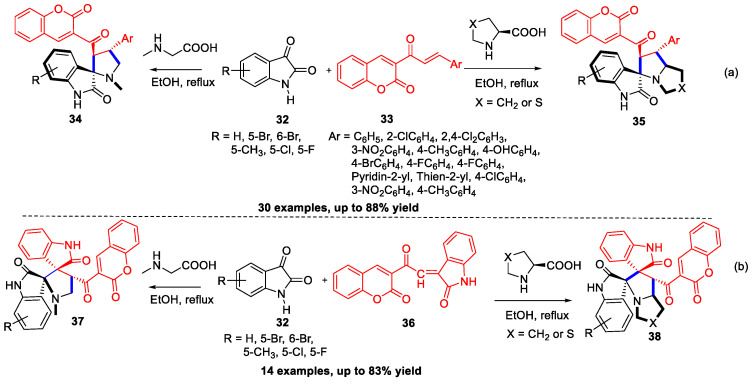
(**a**) A facile and diverse synthesis of coumarin-substituted spiro-oxindole. (**b**) Synthesis of novel coumarin substituted dispiro-oxindole compounds.

In 2021, Zhu et al. accomplished the synthesis of a library of functionalized coumarin ring-grafted spiro or dispiro-oxindole heterocycles via a simple and efficient one-pot three-component 1,3-dipolar cycloaddition reaction involving diverse dipolarophiles and azomethine ylides. This method resulted in the high-efficiency production of a wide range of coumarin-substituted spiro-oxindole and dispiro-oxindole derivatives containing pyrrolizidine/pyrrolothiazole/pyrrolidine moieties (**34**, **35**, **37**, **38**) with high efficiency (Scheme 8). This approach offers several valuable features, notably including high product yields (up to 88%), a broad substrate scope (44 examples), mild reaction conditions, and a straightforward procedure. These characteristics make it an attractive and useful process for synthesizing biologically important compounds [74].

**Scheme 9:**
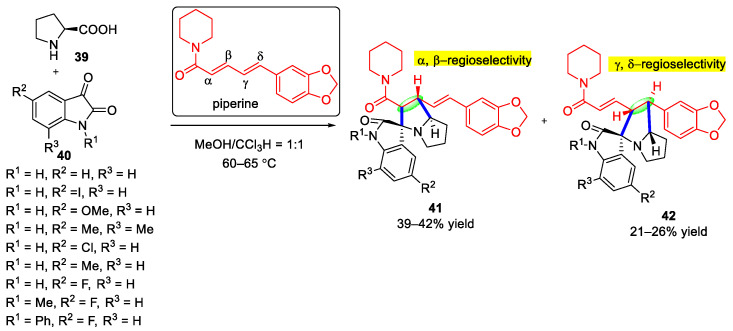
1,3-dipolar cycloaddition with piperine as dipolarophiles.

Piperine (*trans*–*trans*-isomer of 1-piperoyl piperidine), a novel family of alkamides derived from the medicinal plant Piper nigrum (family Piperaceae), has been recognized for its plethora of therapeutic biological activities including anti-inflammatory, anti-arthritic, anti-nociceptive, anti-depression, anti-hypertension, anti-cancer, and antioxidant properties [75,76,77]. In 2019, Hazra and colleagues further investigated the potential of isatin-based 1,3-dipolar cycloaddition by selecting piperine as their compound of choice. They successfully synthesized spiro-oxindole pyrrolizidine adducts through an intermolecular 1,3-dipolar azomethine ylide cycloaddition reaction in a multicomponent (MCR) atom-economical manner. Both double bonds in piperine undergo conjugation and generate two racemic regioisomeric adducts, **41** and **42**, with a higher yield of the former (Scheme 9). The structures of these products were determined by utilizing a combination of 1D/2D NMR, mass spectral analysis, and X-ray crystallography for selected compounds. Additionally, chiral HPLC separation was employed to determine the specific rotation and CD spectra of the enantiomers for two racemic compounds [78].

## 3. Dipolarophiles Containing Exocyclic Unsaturated C=C Double Bonds

**Scheme 10:**
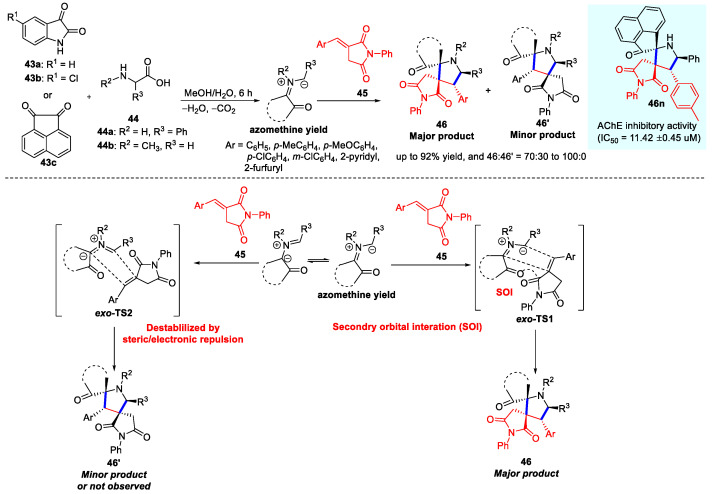
Three-component access to functionalized spiropyrrolidine heterocyclic scaffolds and their cholinesterase inhibitory activity.

In 2020, Boudriga developed an efficient and practical strategy for the multicomponent 1,3-dipolar cycloaddition of (*E*)-3-benzylidene-1-phenyl-succinimides **45**, isatin **43**, and diverse α-amino acids **44**. Under optimal conditions (MeOH/H_2_O, 6 h), succinimide-substituted dispiropyrrolidine derivatives **46** were obtained with high regio- and diastereoselectivities (Scheme 10). The stereochemistry of these *N*-heterocycles has been confirmed by four X-ray diffraction studies. According to the proposed mechanism, the formation of the exo-regioisomer **46** via path A is more favorable because of the presence of a secondary orbital interaction (SOI), which occurs between the oxygen atom of the carbonyl of the diketone and the carbon atom of the carbonyl of the dipolarophile, as shown in Scheme 10. Conversely, the formation of the other regioisomers **46′** is less favorable because of steric or electronic repulsion in their corresponding transition states.

In addition, several synthesized compounds exhibit greater inhibition of acetylcholinesterase (AChE) than butyrylcholinesterase (BChE). Among the 17 tested compounds, 5 compounds demonstrate significant AChE inhibition with IC_50_ values ranging from 11.42 to 22.21 µM. Compound **46n**, which possesses the most potent AChE inhibitory activity (IC_50_ = 11.42 ± 0.45 µM), was subjected to a molecular docking study that revealed its binding to the peripheral anionic site of AChE enzymes (Scheme 10) [79].

**Scheme 11:**
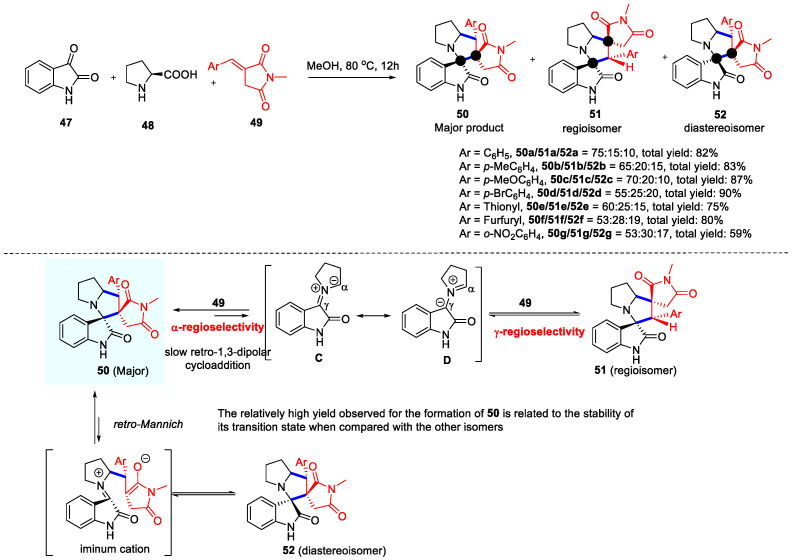
1,3-dipolar cycloaddition reaction involving (*E*)-3-arylidene-1-methyl-pyrrolidine-2,5-diones, L-proline, and isatin.

In 2022, El-Tahawy et al. reported the synthesis of a novel series of spiropyrrolizidine derivatives through a three-component 1,3-dipolar cycloaddition reaction involving (*E*)-3-arylidene-1-methyl-pyrrolidine-2,5-diones **49**, L-proline, and cyclic ketones isatins. Notably, the authors observed that both regioselectivity and stereoselectivity were strongly influenced by solvent properties and reaction time (Scheme 11). Under optimal conditions (MeOH at 80 °C for 12 h), besides the anticipated cycloaddition product **50**, its corresponding regioisomer **51** and diastereoisomer **52** were also synthesized. For the observed epimerization, the authors proposed a ring-opening retro-Mannich reaction for compound **52**, which possesses an unusual relative stereochemistry. Regioisomer **51** formed via cycloadduct **50** could also undergo a slow 1,3-dipolar retro-cycloadduct to generate W-conformal azomethine ylides **D** whose cycloadduct with dipolarophile **49** would lead to the formation of unexpected regioisomers **51**. The proposed mechanism and resulting yields are rationalized by theoretical calculations, and the relatively high yield observed for the formation of **50** is related to the stability of its transition state when compared with the other isomers. Furthermore, synthesized compounds underwent biological activity assays against a variety of microbial strains, revealing moderate to excellent antibacterial, antifungal, and anticoagulant activities [80].

**Scheme 12:**
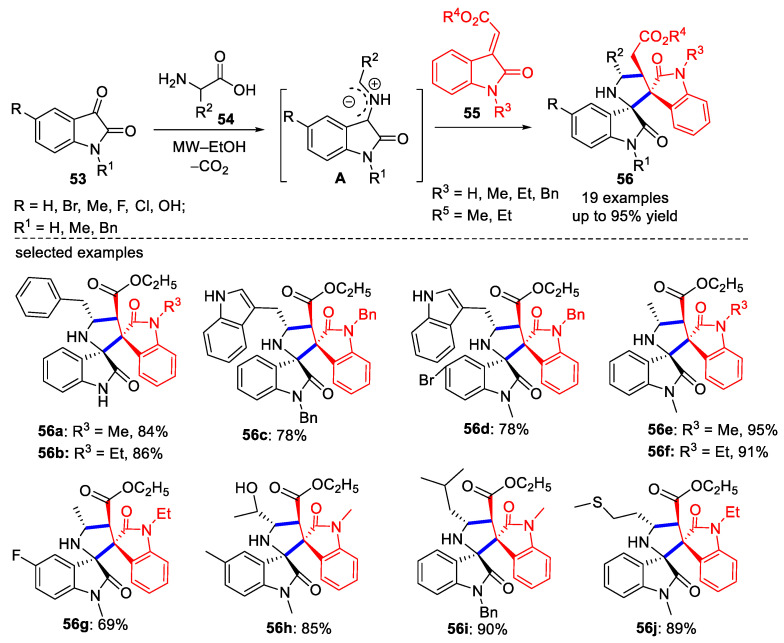
Microwave-assisted one-pot [3 + 2] cycloaddition of azomethine ylides and 3-alkenyl oxindoles.

Bis-spiro-oxindoles display a broad spectrum of biological activities, encompassing anticancer, antiviral, antibacterial, and anti-inflammatory effects [81,82]. These compounds feature two spirocyclic rings fused together, rendering them an intriguing class of organic molecules. Their distinctive structural characteristics combined with their diverse bioactivities render them an appealing synthetic target. In 2019, Shankaraiah developed a diastereoselective and environmentally friendly one-pot protocol for synthesizing pyrrolidine-fused bis-spiro-oxindole **56** using 3-alkenyl oxindoles **55** as substrates (Scheme 12). The reactions were conducted under microwave conditions with ethanol as the solvent, without any additives or catalysts. This method exhibits broad substrate scope (19 examples), high atom economy, and is eco-friendly with excellent yields (up to 95%) [83].

**Scheme 13:**
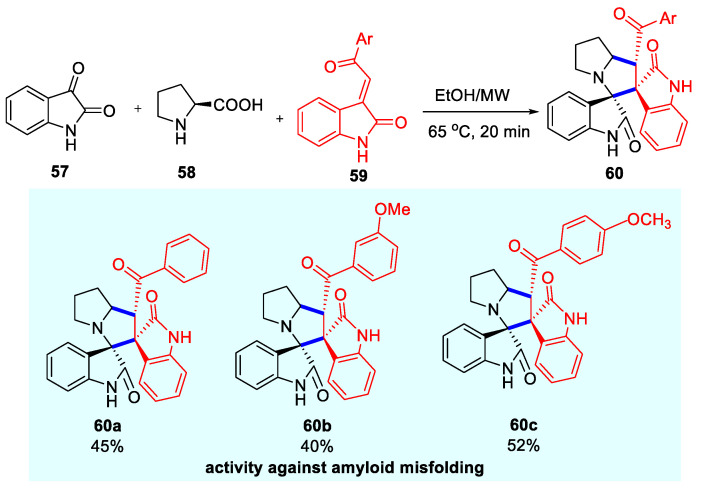
Regioselective synthesis of pyrrolizidine bis-spiro-oxindoles as efficient anti-amyloidogenic agents.

In 2022, Maniam et al. disclosed a rapid microwave-assisted synthesis of novel pyrrolizidine bis-spiro-oxindoles compounds **60a**–**60c** with high stereo- and regiospecificity, eliminating the need for extensive purification procedures (Scheme 13) [84]. Significantly, initial screening of these compounds demonstrated promising activity against amyloid misfolding by actively interacting with highly amyloidogenic regions within the peptide. The advantageous characteristics of this one-pot three-component reaction include facile synthesis and potential anti-amyloidogenic properties.

**Scheme 14:**
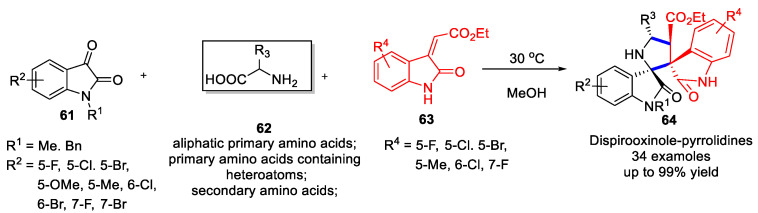
Diastereospecific entry to pyrrolidinyldispirooxindole skeletons via 1,3-dipolar cycloadditions of methyleneindolinones.

Similarly, with MeOH as the solvent and at a temperature of 30 °C, methyleneindolinones **63** could react with isatins and various primary amino acids in the three-component 1,3-dipolar cycloaddition reaction (Scheme 14). Pyrrolidinyl–dispiro-oxindole scaffolds **64** were easily obtained in excellent yields (34 examples and up to 99% yield), exhibiting a wide range of structural diversity and complexity. All amino acid types could smoothly participate in the reaction, with aliphatic amino acids typically yielding excellent results. However, chemical yields were reduced when functional groups such as hydroxyl or imidazole were attached, while diastereoselectivity was maintained. Additionally, this protocol was also applicable to N-substituted analogues of amino acids and exhibits comparable reactivities [85].

**Scheme 15:**
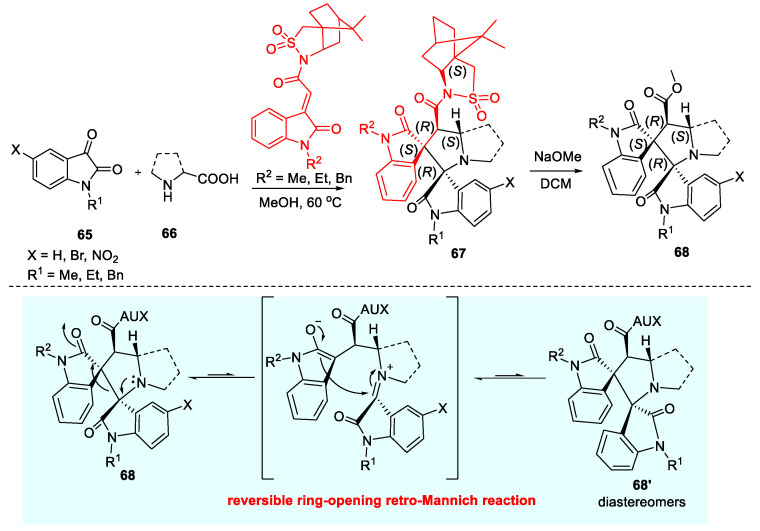
Synthesis of highly enantioenriched bis-spiro-oxindole pyrrolizidine/pyrrolidines through asymmetric [3 + 2] cycloaddition reaction.

In 2020, with (*E*)-2-oxoindolin-3-ylidene acetyl sultam as reaction partner, Jadidi reported the synthesis of a highly enantioenriched bis-spiro-oxindole pyrrolizidine scaffold using 1,3-dipolar cycloaddition of isatin-derived azomethine ylides (Scheme 15). In addition, highly enantioenriched bis-spiro-oxindole pyrrolidines were generated in high yields and with high regio- and -diastereoselectivities (up to 99:1) by utilizing this chiral auxiliary controlled method. It should be noted that these products were completely stable in a solid state. However, when the pure samples were kept as a solution in room temperature, the other diastereomers **68′** were gradually observed after about 2 h. The author speculated that these obtained cycloadducts underwent retro-Mannich ring-opening cyclization and produced new diastereoisomers of the bis-spiro-oxindole pyrrolizidine/pyrrolidines skeleton, which could not be accessed by direct 1,3-dipolar cycloaddition [86]. 

**Scheme 16:**
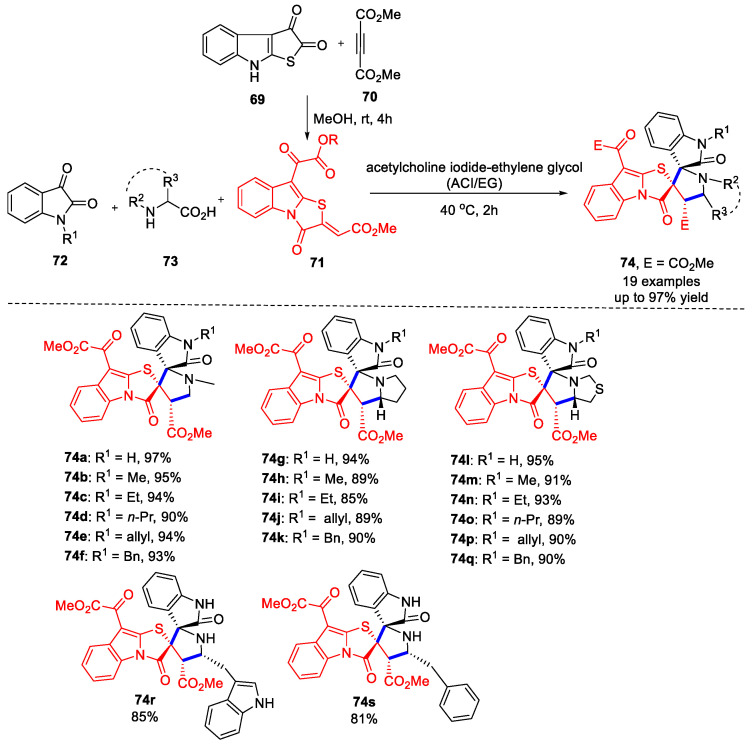
[3 + 2] cycloaddition of azomethine ylides with a thiazolo[3,2-*a*]indole derivative.

An intricate polycyclic dipolarophile **71** was synthesized via the reaction of thieno[2,3-b]indole-2,3-dione **69** and dimethyl acetylenedicarboxylate **70** in MeOH at ambient temperature. With the compound **71** in hand, dispiro-heterocyclic thiazolo[3,2-a]indole derivative **74** was obtained through [3 + 2] cycloaddition reactions of compound **71** with azomethine ylides (Scheme 16). Notably, an eco-friendly deep eutectic solvent system composed of acetyl choline iodide–ethylene glycol (ACI/EG) was employed for the reaction. Spiropyrrolidine oxindoles with multiple stereocenters were obtained in a highly diastereoselective manner and excellent yields (19 examples, up to 97% yield) [87].

**Scheme 17:**
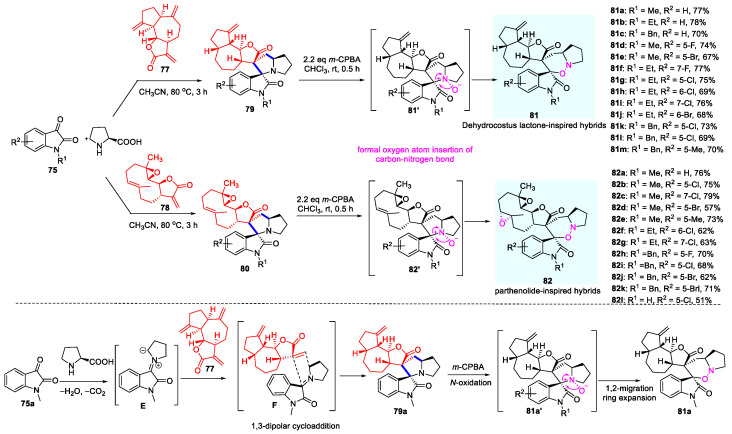
Synthesis of dehydrocostus-lactone-inspired hybrid and parthenolide-inspired hybrid via formal oxygen atom insertion.

Developing effective strategies for single-step editing of the core skeleton of structurally complex compounds is a highly desirable yet challenging task. Dehydrocostus lactone and parthenolide are heterocyclic natural products of significant importance, as their biological activity may be influenced by their stereochemistry and substituents [88,89,90,91]. In 2023, Liu et al. accomplished the synthesis of a structurally intriguing dehydrocostus-lactone-inspired hybrid **79** via a 1,3-dipolar cycloaddition of isatin **75**, proline and optically pure dehydrocostus lactone **77**. Subsequently, in the presence of *m*-CPBA, they made an unexpected discovery that enabled bispiro[oxindole-oxazinane] skeletal editing of a pyrrolidine skeleton to a 1,2-oxazinane skeleton based on formal oxygen atom insertion into a carbon–nitrogen bond (Scheme 17). These structurally diverse and medicinally important compounds, which contain up to six adjacent stereocenters including two quaternary spiro-stereocenters, were synthesized in just two steps with excellent yields (up to 79% yield and >20:1 dr). Similarly, the synthesis of bispiro[oxindole-oxazinane] hybrids **82** inspired by parthenolide could be achieved using the same method.

The authors also provided the proposed mechanism (Scheme 17). First, the reaction of isatin **75a** and proline led to azomethine ylide **E** via a dehydration/decarboxylation process, which could be added to optically pure dehydrocostus lactone **77** in a sequential manner to give cycloaddition compound **79a**. Subsequently, *N*-oxidation with *m*-CPBA would lead to the formation of pyrrolidine-derived *N*-oxide skeleton **81a′**. However, **81a′** was unstable, perhaps due to the unfavorable repulsion between three adjacent tetra-substituted stereocenters, leading, subsequently, to the oxygen atom in the *N*-oxide group attacking the C3 spiro center of oxindole via a 1,2-migration process followed by a ring expansion to access the unexpected 1,2-oxazinane skeleton **81a**. This protocol represents a significant advancement in skeletal editing, as it is the first example of converting a pyrrolidine skeleton to a 1,2-oxazinane skeleton, thereby expanding the diversity of spiro-oxindoles [92].

## 4. Dipolarophiles Containing Endocyclic Unsaturated C=C Double Bonds

**Scheme 18:**
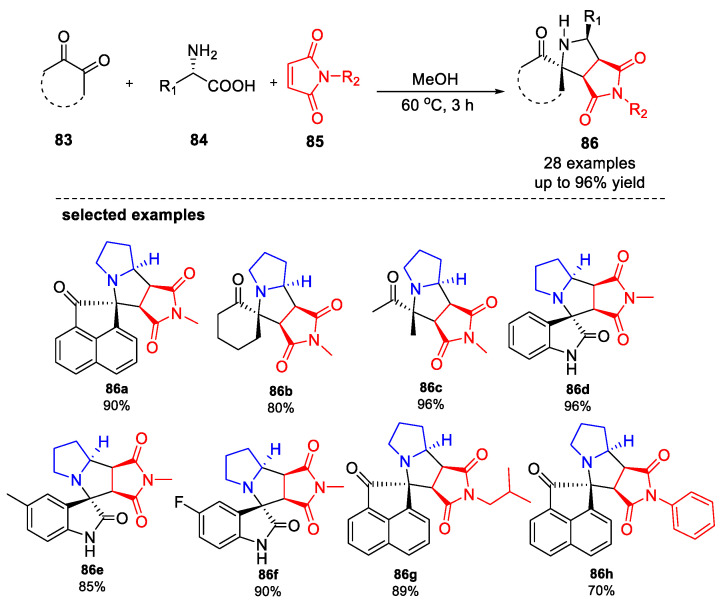
A three-component reaction of α-diketone, amino acid, and maleimide.

Based on our extensive interest in maleimide chemistry [93,94], our research group has successfully developed an efficient one-pot, three-component [3 + 2] cycloaddition reaction of azomethine ylides derived from α-dicarbonyl compounds (isatins, diketones, or keto esters) and amino acids with maleimides under mild conditions (Scheme 18). The cascade protocol demonstrates high efficiency and remarkable tolerance towards functional groups (28 examples), resulting in the formation of widely distributed succinimide-fused pyrrolizidines with a highly compact and strained scaffold, achieving high yield and excellent diastereoselectivity (up to 96% yield and >20:1 dr) [95].

**Scheme 19:**
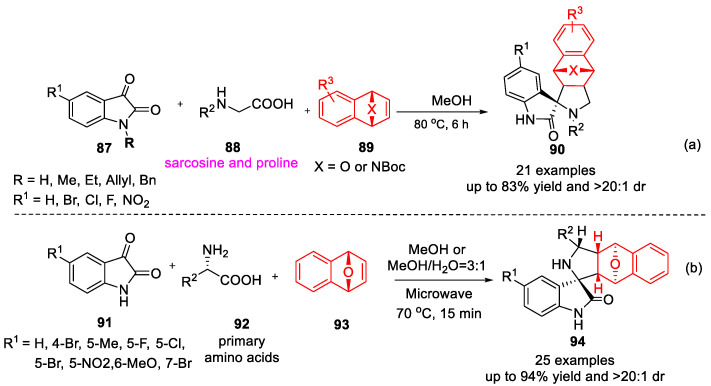
(**a**): 1,3-Dipolar cycloaddition of azomethine ylides with heterobicyclic alkenes. (**b**): Synthesis of oxygen-bridged spirooxindoles via microwave-promoted multicomponent reaction.

Oxabicyclic alkenes, due to their inherent strain, exhibit intriguing reactivities and have been widely applied in ring-retentive and ring-opening catalytic transformations, including dimerization [96], cycloaddition [97], ring-opening/rearrangement reactions [98], hydrofunctionalization [99,100,101,102], and C–H activation [103,104]. Although significant progress has been achieved in the conventional 1,3-dipolar cycloaddition of azomethine ylides derived from isatins and α-amino acids with diverse electron-deficient alkenes, the involvement of strained oxabicyclic alkenes remains limited. Parthasarathy and co-workers explored 1,3-dipolar cycloaddition of azomethine ylides with heterobicyclic alkenes **89** at 80 °C for 6 h (Scheme 19a), and various fused spiropyrrolidine oxindoles were isolated in good yield [105]. Our group noticed that the amino acids substrate scope was limited in sarcosine and proline. Subsequently, our research group investigated an environmentally friendly microwave-assisted multicomponent reaction involving isatins, α-amino acids (primary and secondary), and 1,4-dihydro-1,4-epoxynaphthalene (Scheme 19b). This reaction resulted in the formation of oxygen-bridged spiro-oxindoles **94** with high yields ranging from good to excellent (up to 94%) within a remarkably short time of only 15 min [106].

**Scheme 20:**
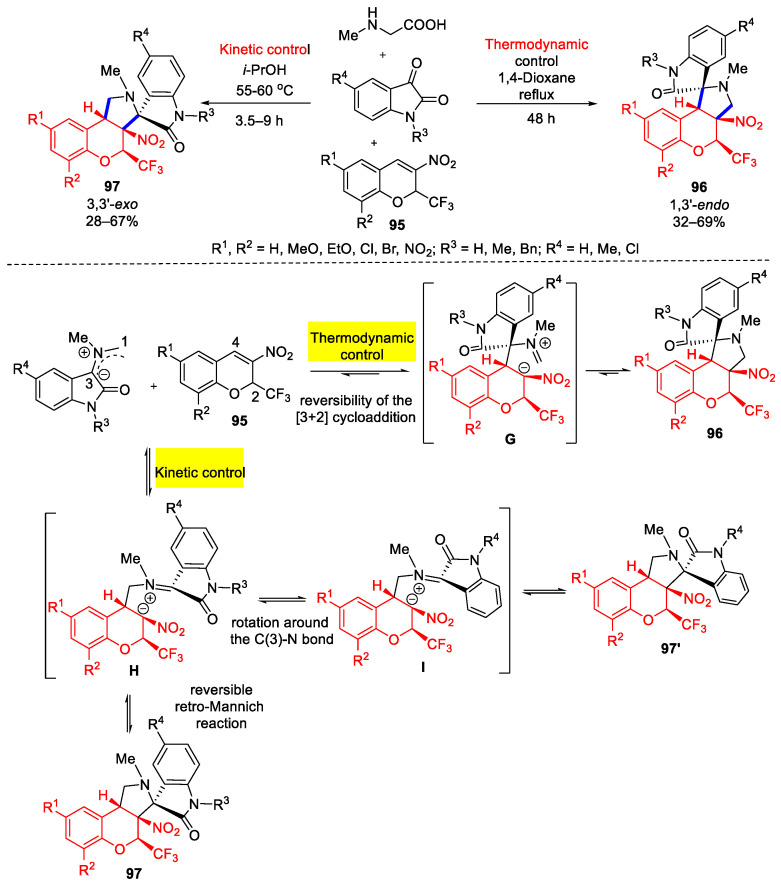
A reversible [3 + 2] cycloaddition of azomethine ylides with 3-nitro-2-(trifluoromethyl)-2*H*-chromenes.

Azomethine ylides generated in situ from isatins and sarcosine with 3-nitro-2-(trifluoromethyl)-2*H*-chromenes **95** in *i*-PrOH at 55–60 °C lead to the kinetically controlled products exo-spiro[chromeno[3,4-c]pyrrolidine-3,30-oxindoles] **97**. When this reaction is carried out in 1,4-dioxane under reflux, the thermodynamically controlled endo-spiro[chromeno[3,4-c]pyrrolidine-1,30-oxindoles] **96** are preferably formed (Scheme 20). From a mechanistic perspective, due to the high electrophilicity of the double bond, several [3 + 2] cycloaddition reactions of 1,3-dipoles to nitroalkenes proceed via a nonconcerted Michael addition/Mannich reaction sequence. The introduction of the azomethine ylide to chromene **95** results in the formation of zwitterionic intermediates **G**, which subsequently undergo cyclization under thermodynamically controlled conditions to yield the corresponding products **96**. However, it should be noted that this reaction is reversible and may result in the regeneration of the starting material. In contrast, under kinetic control, the dipole attacks the C-4 atom of chromene **95** via the less substituted C-1 atom, while under thermodynamic control, addition of ylide to chromene through the C-3 atom is more favorable. In addition to the kinetic control product **97**, the zwitterionic intermediate **H** can be transformed into intermediate **I** by rotation around the C (3)-N bond, yielding another diastereomer **97′**. Moreover, the study also investigated the cytotoxicity of certain spiro[chromeno-[3,4-c]pyrrolidine-oxindoles] substituted with CF_3_ against the HeLa cell line using the MTT assay [107].

**Scheme 21:**
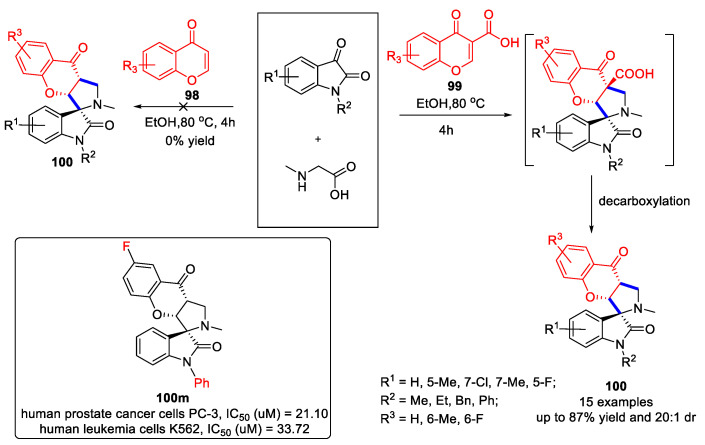
The construction of chromanone-fused pyrrolidinyl spiro-oxindole collections through a decarboxylative 1,3-dipolar [3 + 2] cycloaddition reaction.

The heterocycle-based chromanone structures are considered privileged skeletons for their diverse biological activities in natural products [108,109,110]. In 2019, Liu et al. developed a novel approach for the facile one-pot multicomponent synthesis of heterocycle-fused spiro compounds **100**, which integrated two potential pharmacophores, chromanone and pyrrolidinyl spiro-oxindoles, into a single molecule (Scheme 21). In contrast, the absence of the carboxylic acid group rendered **98** incapables of generating the desired cycloaddition product **100**. A wide variety of products with three contiguous stereocenters were smoothly prepared with high efficiency (up to 87% yield and 20:1 dr). The compound obtained exhibited biological activities against human prostate cancer cells PC-3 and human leukemia cells K562; notably, the compound **100m** exhibited IC_50_ values of 21.10 μM and 33.72 μM for the two tested targets, respectively [111].

**Scheme 22:**
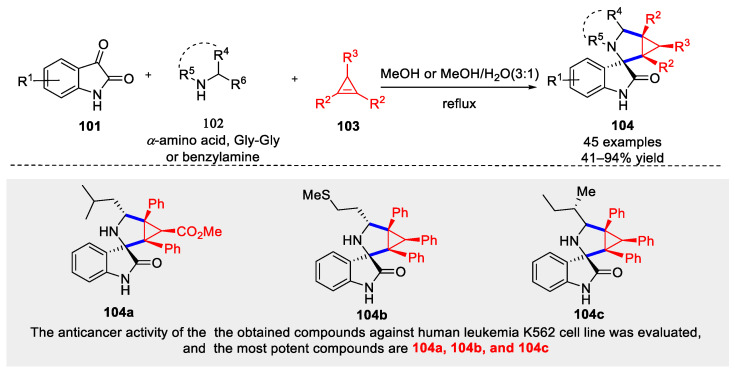
The [3 + 2]-cycloaddition of cyclopropenes and azomethine ylides.

Cyclopropenes are fascinating molecules that have captured the attention of chemists for decades [112,113,114]. The smallest possible unsaturated carbocycle, with their characteristic triangular shape, owes an incredible reactivity to 54.1 kcal/mol of strain energy. Releasing the strain energy enables cyclopropenes to undergo various reactions such as cycloadditions and hydrofunctionalizations [115,116,117,118]. In 2017, Stepakov and co-workers disclosed a one-pot three-component reactions utilizing substituted isatins, α-amino acids, and cyclopropenes **103** (Scheme 22). It is noteworthy that both dipeptide Gly–Gly and benzylamine can serve as the amine component for azomethine ylide generation. The in vitro anticancer activity of some of the obtained compounds against human leukemia K562 cell line was assessed using flow cytometry [119].

## 5. Dipolarophiles Containing Unsaturated C=N Double Bonds

**Scheme 23:**
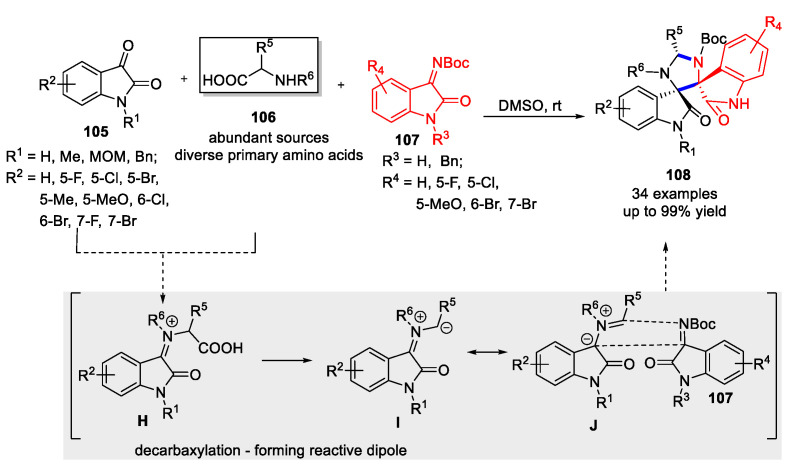
A diversity-driven three-component 1,3-dipolar cycloaddition of isatins, amino acids, and isatin-derived ketimines.

In terms of dipolarophiles of 1,3-dipolar cycloaddition, a considerable number of alkenes and alkynes have been subjected to 1,3-DC leading to the formation of various spiro-oxindolopyrrolidines and spiro-pyrrolines. On the other hand, the synthesis of analogous spiro-oxindoloimidazolidines using imines as dipolarophiles remains largely unexplored. Yang and co-workers developed a diversity-driven three-component 1,3-dipolar cycloaddition of isatins, amino acids, and isatin-derived ketimines **107** (Scheme 23). A variety of dispiro-oxindole–imidazolidine frameworks featuring adjacent quaternary carbon centers were facilely obtained in excellent yields under mild conditions (at room temperature). This protocol can be readily scaled up with simplified purification steps without compromising the chemical outcome. The broad substrate scope (34 examples) and high chemical yield (up to 99%) rendered this strategy particularly appealing for expanding the library of dispiro-oxindoles [120].

**Scheme 24:**
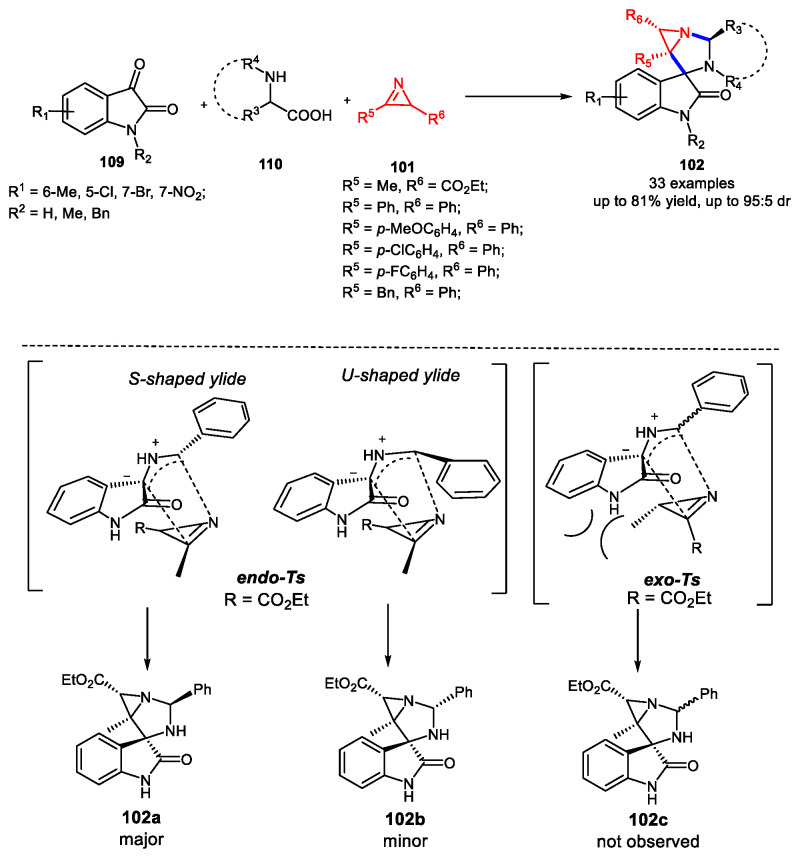
1,3-Dipolar cycloaddition of isatin-derived azomethine ylides with 2H-azirines.

The incorporation of 2H-azirines as dipolarophiles in cycloaddition reactions involving isatin-derived azomethine ylides has been explored by Kanizsai and co-workers, which presents a fascinating opportunity to investigate the potential reactivity and versatility of these compounds. They elaborated a regio- and diastereoselective 1,3-dipolar cycloaddition of 2*H*-azirines with azomethine ylides generated in situ from isatins and α-amino acids, affording an unprecedented aziridine-fused spiro[imidazolidine-4,3′-oxindole] framework (Scheme 24). This one-pot three-component reaction exhibits broad substrate tolerance (33 examples) and enables facile construction of highly diverse 1,3-diazaspiro[bicyclo[3.1.0]hexane]oxindoles under mild conditions with isolated yields up to 81%. Mechanistically, the reaction between azomethine ylides and 2H-azirine can proceed via both endo-transition state (TS) and exo-transition state (TS); no evidence was discovered regarding the formation of exocyclic products. The exclusive formation of endo-cycloadducts **102a** and **102b** may be attributed to steric repulsion between the methyl substituent of the azirine moiety and the phenyl substituent of oxindole in exo-TS. Due to its more favorable *S*-shaped conformation over *U*-shaped conformation, endo-selective 1,3-DC leads to the predominant formation of diastereomer **102a** [121].

## 6. Other Dipolarophiles

**Scheme 25:**
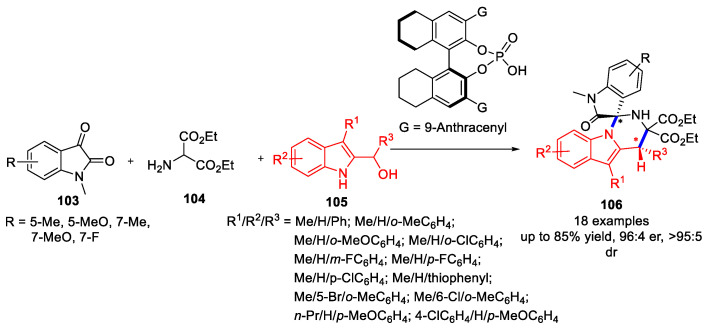
Diastereo- and enantioselective construction of spiro-oxindole scaffolds through a catalytic asymmetric [3 + 3] cycloaddition.

The versatility of 2-indolylmethanols as reactants in the synthesis of indole derivatives or indole-fused cyclic frameworks has been well-documented [122,123,124,125]. However, there is a limited number of examples showcasing catalytic enantioselective cycloadditions utilizing 2-indolylmethanols as nitrogen–carbon–carbon (NCC) or three carbon (3C) building blocks. The chiral phosphoric acid-catalyzed enantioselective and regioselective [3 + 3] cycloaddition of isatin-derived azomethine ylides was conducted in 2017, employing C3-unsubstituted 2-indolylmethanols as NCC building blocks (Scheme 25). This reaction afforded chiral spiro-oxindoles with significant yields, displaying moderate to good enantioselectivities and excellent diastereoselectivities (up to 85% yield, 96:4 er, all > 95:5 dr) [126].

**Scheme 26:**
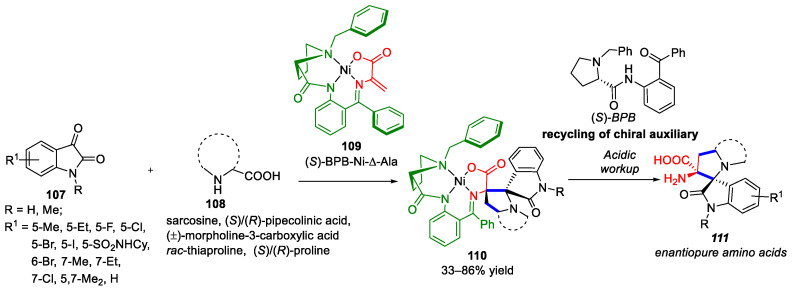
An asymmetric 1,3-dipolar addition of azomethine ylides to a chiral dehydroalanine Ni(II) complex.

Chiral unnatural amino acids (AAs) are highly sought after in the fields of biochemistry and pharmaceuticals due to their extensive applications for peptide and protein modification, enhancement of metabolic stability, reduction in aggregation, improvement of pharmacokinetic properties, and facilitation of blockbuster drug design [127,128,129,130]. The Ni(II) complex is a widely recognized and versatile tool for the synthesis of challenging enantiopure amino acids. Notably, the chiral auxiliary released after isolation of the amino acid can be conveniently recovered through precipitation and reused in the synthesis of the initial Ni(II) complex. In 2022, Larionov and co-workers reported a protocol for the asymmetric synthesis of artificial amino acids featuring a 3-spiropyrrolidine oxindole skeletal with continuous tetra-substituted carbon stereocenters (Scheme 26). This was achieved through a 1,3-dipolar cycloaddition reaction of in-situ-generated azomethine ylides with a chiral dehydroalanine Ni(II) complex **109**. The quantum chemical calculation was employed to elucidate the formation of distinct regioisomers in sarcosine and proline. The acidic decomposition of Ni(II) complexes **110** afforded the desired unnatural complex AAs **111** featuring a 3-spiropyrrolidine oxindole core. The chiral auxiliary ligand was reclaimed and employed for the synthesis of the initial dehydroalanine complex–substrate [131].

## 7. Conclusions

The one-pot 1,3-dipolar cycloaddition reactions have emerged as a prominent research area in the field of multicomponent reactions, garnering significant attention from both organic and medical chemistry communities. Multicomponent one-pot 1,3-dipolar cycloaddition reactions offer a versatile approach for synthesizing natural products, heterocyclic scaffolds, medicinal drug molecules, and functional materials. Among them, the in situ generation of azomethine ylides from isatins and amino acids with dipolarophiles represents the most fascinating strategy. The abundance and easy availability of starting materials enable the construction of a diverse library of spiro-oxindole scaffolds. In addition to azomethine ylides, a wide range of dipolarophiles including unsaturated C=C bonds with electron-withdrawing groups such as cyano, tosyl, ketone, ester, carboxylic acid, nitro, and amide can be effectively utilized in diversity-oriented reactions. However, it is worth noting that there continues to be a scarcity of more intricate dipolarophiles, and experts may propose innovative alternative components for constructing structures with potential medical applications. On the other hand, devising effective strategies for asymmetric catalytic synthesis and the manipulation of product core skeletons remains an immensely desirable yet formidable task.

## Data Availability

Data sharing not applicable.

## Sample Availability

Samples of the compounds are not available from the authors.
